# Effect of Honey Bee Venom on Lewis Rats with Experimental Allergic Encephalomyelitis, a Model for Multiple Sclerosis

**Published:** 2012

**Authors:** Akbar Karimi, Farhad Ahmadi, Kazem Parivar, Mohammad Nabiuni, Saied Haghighi, Sohrab Imani, Hossein Afrouzi

**Affiliations:** a *Department of Biology, Science and Research Branch, Islamic Azad University, Tehran, Iran. *; b *Department of Toxicology and Pharmacology, Faculty of Pharmacy, Shahid Beheshti University of Medical Sciences, Tehran, Iran.*; c *Tarbiat Moallem University, Tehran, Iran.*

**Keywords:** Bee venom, Multiple sclerosis, Experimental allergic encephalomyelitis, TNF-α, Serum nitrate

## Abstract

Multiple sclerosis (MS) is a progressive and autoimmune neurodegenerative disease of the central nervous system (CNS). This disease is recognized through symptoms like inflammation, demyelination and the destruction of neurological actions. Experimental allergic encephalomyelitis (EAE) is a widely accepted animal model for MS. EAE is created in animals by injecting the tissue of myelin basic protein (MBP), CNS, or myelin oligodendrocyte glycoprotein (MOG) along with the adjuvant. EAE and MS are similar diseases. Honey Bee venom (*Apis mellifera*) contains a variety of low and high molecular weight peptides and proteins, including melittin, apamin, adolapin, mast cell degranulating peptide and phospholipase A2. Bee venom (BV) could exert anti-inflammatory and antinociceptive effects on the inflammatory reactions. The guinea pig spinal cord homogenate (GPSCH) is with the Complete Freund’s Adjuvant (CFA), consisting of 1 mg/mL *Mycobacterium tuberculosis.* It was used for inducting EAE in Lewis rats for creating the MS model.

The hematoxylin and eosin and luxol fast blue methods were used respectively in analyses of inflammation and detection of demyelination in the central nervous system. Furthermore, the ELISA and the high performance liquid chromatography (HPLC) were used for the assessment of tumor necrosis factor alpha (TNF-*α*) and nitrate in rats serum. In this study, we indicated that the treatment of EAE with Bee venom decreased the symptoms of clinical disorder, pathological changes, inflammatory cell infiltration, demyelination in the central nervous system, level of serum TNF-*α*, and the serum nitrates in rat EAE induced through GPSCH.

## Introduction

Most scientists would describe multiple sclerosis (MS) as an autoimmune disease that affects the central nervous system (CNS) and it is recognized through symptoms like inflammation, demyelination and the destruction of neurological actions (1, 2). Experimental allergic encephalomyelitis (EAE) is considered as a valuable animal model for MS research and the scientists that use it both for the evaluation of the process and treatment of diseases (3). EAE is induced in animals by injecting the tissue of MBP or MOG along with the adjuvant. EAE and MS are similar diseases. In EAE, the certain symptoms such as paralysis, inflammation, ataxia, elevated interferon-*γ* (IFN-*γ*), brain-blood, barrier damage and the penetration of CD4^+^ T-cells and macrophages to the central nervous system are observed (4, 5).

Therefore, MS is complex and heterogeneous. Its pathogenesis remains unknown and specific effective treatment has not been developed. Thus, the investigation for alternative medications continues and scientist searches many complementary and alternative medicines (CAM) for patients. Among the CAM therapies, the medication apogean has been developed from venoms (such as: snakes, scorpion and honey Bee) (6).

The venom of honey Bee (*Apis mellifera)* consists of different types of light and heavy chain peptides and the proteins such as melittin, apamin, adolpin and phospholipase A2. Moreover, anti-inflammatory specifications of Bee venom with induced arthritis have been reported in the rat model. It is observed that the injection of Bee venom suppresses the leukocyte migration and reduces the level of TNF-*α* (6). The healing Bee poison is largely effective in treating the chronic anti-inflammatory diseases (7). Bee venom contains a variety of peptides, including melittin, apamin, adolapin, the mast-cell-degranulating (MCD) peptide, enzymes, biologically active amines, and nonapeptide components, which have a variety of pharmaceutical properties. These properties of BV leads to its traveling along the neural pathways from the spine to various trigger points and injury areas to repair the nerve damage and restore the mobility (8).

In this experiment, we study anti-inflammatory effects of honey Bee venom and effects of honey Bee venom on rat with EAE to investigate the Bee venom for the treatment of MS.

## Experimental

The Iranian Honey Bee venom (*Apis mellifera*) was prepared through placing Bees on a 6 mm wire grid, which was electrically pulsed. The Bees then produced venom that dropped onto a glass slide, which was collected from the glass and freeze dried. Complete Freund’s adjuvant purchased from Sigma-Aldrich. The TNF-*α* ELISA Kit is purchased from Abcam. Ketamine and xylazine are purchased from Alfasan and Holand. Other chemicals were of analytical grade and purchased from Merck.

EAE was induced according to the method of Shnider* et al. *(2009), via subcutaneous injection of 0.2 mL GPSCH which was emulsified in 1:1 ratio of complete F adjuvant (GPSCH-CFA) to the adult female Lewis rats (weight, 180-200 g; Laboratory of Animal Center, Darupakhsh Pharmaceutical Company, Tehran, Iran) (9).

The animals used in this research were kept under the standard conditions and fed with water and food ad-libitum. The experimental procedures were done in accordance with the Guide for the Care and Use of Laboratory Animals published through the National Academy Press, which was accepted by the ethnic committee of the AUSR in Iran (Washington D.C. 1996).

EAE was induced in 30 rats randomly placed in three groups of 10: Group 1: Named E-S, received normal saline (0.2 mL) every day. Group 2: Named E-BV1, received 2 mg/Kg honey Bee venom every day. Group 3: Named E-BV2, received 5 mg/Kg honey Bee venom every day.

The treatments started from the first day of post immunization through GPSH-CFA and lasted until the tenth day.

The day of GPSH-CFA injection was considered as the zero day of post immunization (dpi). Rats were evaluated daily for any unwanted symptoms and weight changes. Then, they were scored daily through the following degrees: 0: Normal and without symptoms. 1: Tail without natural stretch. 2: Paralysis of the tail. 3: Partial paralysis in hind legs. 4: Complete paralysis of hind legs. 5: Tetraplegia. 6: Death.

Rats were anesthetized after injecting the combination of ketamine and xylazine. Then, their brain and spinal cords were removed. They were kept under the process of histotechnique for 24 h in 10% formalin as a fixative*.* The sections of brain and spinal cord were then stained with hematoxylin and eosin (H and E) for inflammatory cell infiltration and luxol fast blue (LFB) for demyelination analyses.

The intensity of inflammatory cell infiltration was assessed according to the protocol of Okuda *et al.*, (1999) and classified according to the obtained scores as follows: 0: The absence of inflammation.1: The penetration of cells around the blood vessels and meninges. 2: Subtle penetration of cells in the parenchyma (1-10/section). 3: Average penetration of inflammatory cells in the parenchyma (1-100/section) (10).

The rate of serum TNF-*α* was specified by using a rat TNF-*α* ELISAKIT. The rate of serum nitrate was specified through the method of HPLC according to Xia *et al.* (2003) (11).

**Figure 1:**
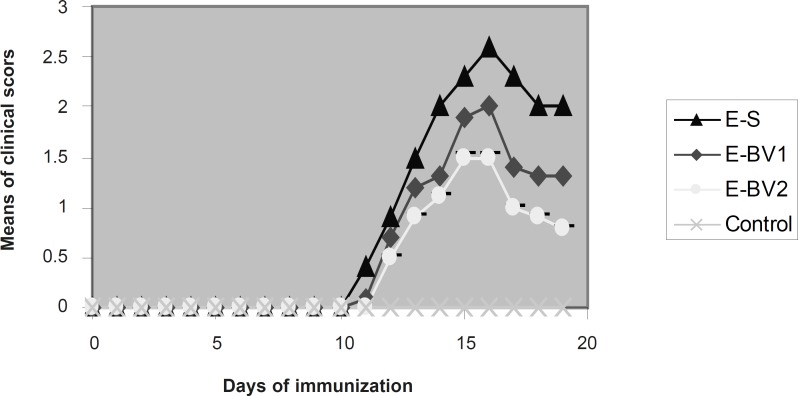
Effect of Bee venom on the clinical score in EAE rats induced by GPSCH–CFA. The onset of clinical signs of EAE was seen at 11 dpi. After five days, the average of clinical scores reached maximum and then it was reduced. Bee venom caused a considerable reduction in the maximum rate of the average clinical scores compared to the group receiving normal saline.

Data were analyzed using the SPSS statistical program (version 17 for Windows). In all the cases for comparison between different groups, Mann-Whitney U-test was used. Significance level was set at p < 0.05 throughout the experiment. Cronbach›s *α* (alpha) was used for validity and reliability test, and R^2^ for correlation test.

## Results and Discussion

Following the immunization of the rats with GPSCH-CFA, some of them in different groups from nine dpi, showed the signs of decreased search activities, nutrition behavior and weight loss. On 11 dpi, the signs like tail stretch loss and partial paralysis of the tail were seen in E-S group. These signs were seen in the E-BV group on 12 dpi and in the E-BB group with 13 dpi and were increased day by day, which finally resulted in the complete paralysis of the hind legs. These sings were observed in great amounts within the E-S group (Figure 1). The average intensity of diseases in E-BV1 and E-BV2 groups were decreased considerably in comparison with the E-S group. Results show that honey Bee venom can meaningfully decrease the clinical symptoms and effects of immunization of Lewis rats with GPSH-CFA.

**Figure 2:**
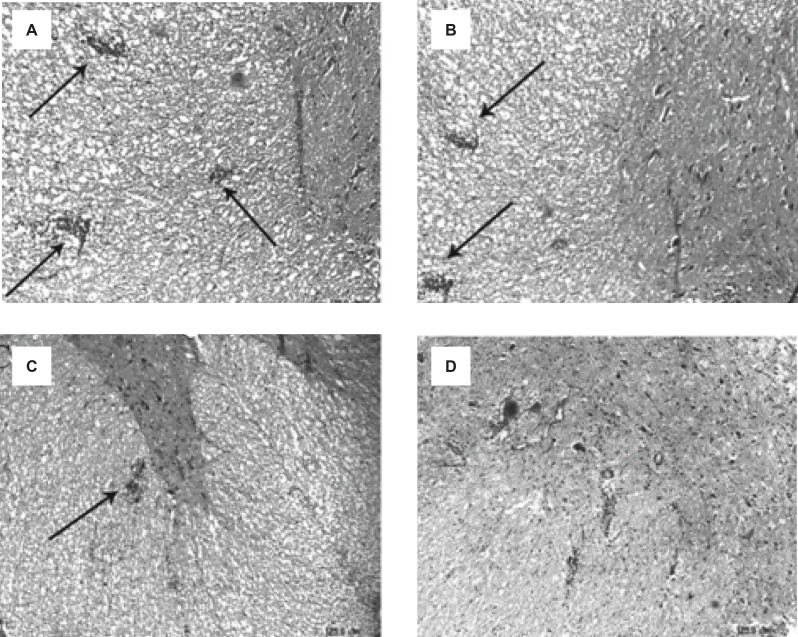
Reduction of the inflammatory cells penetration in the spinal cords of EAE rats, induced by GPSCH-CFA, after the administration of Bee venom (A) E-S group, (B) E-BV1 group, (C) E-BV2 group and (D) Control group (Scale bar A-D = 125 µm).

**Figure 3:**
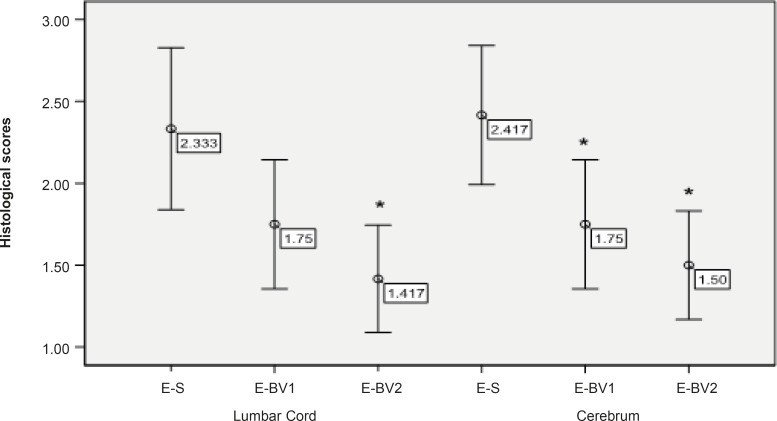
Reduction of the inflammatory cells penetration in the cerebrum and lumbar cords of EAE rats, induced by GPSCH-CFA, after the administration of Bee venom. Pathological changes in rats were assessed by using the semi-quantitative score as described in the Experimental Procedure section (*p < 0.05) compared to E-S group, where n = 4 for all groups.

Following the staining sections and analyzing the samples with the light microscope, no penetration of inflammatory cells of brain parenchyma and spinal cord was observed in the control group tissue samples, while, in samples containing the signs of penetration of mononuclear inflammatory cells in parenchyma, the existence of inflammatory cells around the blood vessels and meninges belonged to three labeled groups. These signs were observed with varying intensity in the specified groups. The intensity of pathological changes and the penetration of inflammatory cells, both in the brain tissue and spinal cord of E-S group, was noticeable. The intensity of pathological changes in the groups of E-BV1 and E-BV2 showed a significant decrease (Figures 2 and 3). The findings of received scores in four labeled groups were in accordance with the results of clinical investigations. In this investigation, Bee venom caused a decrease in penetration of inflammatory cells with observed pathological changes.

**Figure 4:**
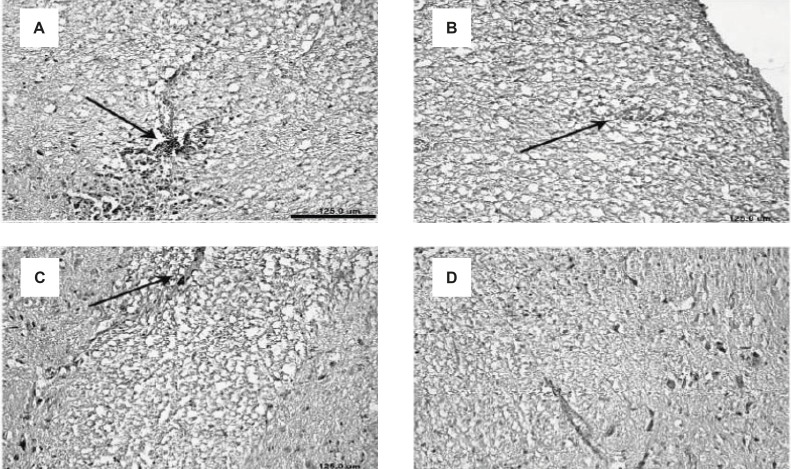
Decreased demyelination of the CNS in EAE rats, induced by GPSCH-CFA, after the treatment with Bee venom. A-D displays the slices of spinal cord and their demyelination process within different experimental groups: (A) E-S group, (B) E-BV1 group, (C) E-BV2 group and (D) Control group (Scale bar A-D = 125 µm).

Along with the results of the inflammatory cell penetration, demyelination showed a decrease for the treatment groups. Demyelination was not observed in the control group but this process was considerably increased in the E-S group. Demyelination in the E-BV2 group showed a significant decrease as compared to the E-S group (Figure 4). These results have shown that Bee venom can considerably decrease the demyelination process, which is caused by the administration of GPSCH-CFA vaccine to the rats.

**Figure 5:**
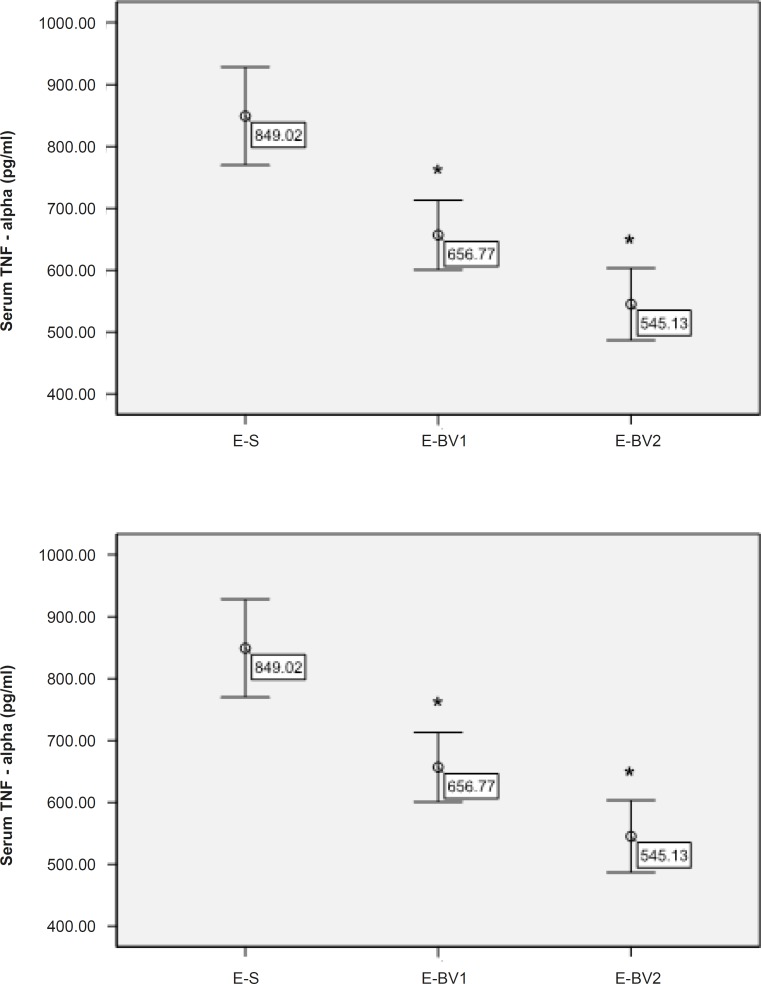
Reduction of the serum TNF-*α* in the rats, induced by GPSCH-CFA, after the treatment with Bee venom. ELISA was used for the determination of serum TNF-*α*. The level of TNF-*α* in different groups compared to E-S, where *p < 0.05 in all the treatment groups.

The amount of TNF-*α* in the serum of rats was measured through ELISA method for different treatment groups. The amount of TNF-*α* had been decreased in the treatment groups compared to E-S and this reduction process in the group E-BV2 was significant (Figure 5). These results have demonstrated that Bee venom causes a decrease in the amount of serum TNF-*α*, which was considerably affected by GPSCH-CFA.

**Figure 6:**
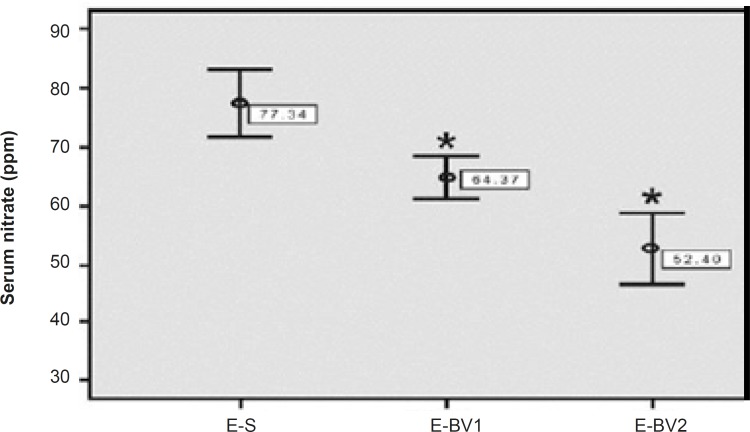
Reduction of serum nitrate levels in the rats, induced by GPSCH-CFA, after the treatment with Bee venom. HPLC was used for the determination of nitrate. The level of nitrate in different groups compared to E-S, where *p < 0.05 in all the treatment groups.

The amount of serum nitrates was evaluated for the treatment groups with HPLC method in order to consider the antioxidant and anti-inflammatory effects of Bee venom. The amount of serum nitrates in E-S group had been increased considerably as compared to the control group. This amount showed a significant decrease in the E-BV1 and E-BV2 groups compared to the E-S group (Figure 6). The results showed that Bee venom can decrease the amount of serum nitrates, which were induced by GPSCH-CFA.

Multiple sclerosis (MS) is an autoimmune disease of CNS, which shows pathological characteristics like the penetration of macrophages and lymphocytes into the CNS, demyelination and axonic damage and, *etc*. Etiology of this disease is unknown, but in this disorder, the myelinated parts of CNS are attacked by T and B cells (12, 13).

Medicinal properties of Bee products have been known from ancient times and today the Bee venom is used extensively for the treatment of arthritis and other inflammatory, autoimmune and destructive diseases (7). Bee venom includes some kinds of peptides, enzymes, active amines and other components, which can be effective in the treatment of various diseases. For example, melitin is one of the most effective and well-known anti-inflammatory factors. Adolapin is another effective anti-inflammatory substance that suppresses the activity of cyclooxygenase (COX) enzyme (8). It is reported that Bee venom prevents the production of Interleukin-1*ß* (IL- 1*ß*) from macrophages in rats in response to the inner stimulation by bacterial lipopolysaccharides (14).

Primary allergic compounds of Bee venom such as histamine and phospholipase A2 induced the production of Interleukin-10 (IL-10) through the T-helper 2 (Th2) cells, suppress T-cell proliferation and can be effective in the reduction of inflammation and demyelination (15).

Bee venom causes a reduced expression of matrix metalloproteinase 2 and 9 (MMP2 and MMP9) (16). MMP2 and MMP9 are related to MS disease and their amount increases in EAE condition (17).

The role of Bee venom in increasing interferon-*ß* (IFN-*ß*) level is a well understood phenomenon (16). According to the study carried out by Mastronardi *et al.* (2004), it was seen that the combination of IFN-*ß* and vitamin B12 led to a significant reduction of clinical and pathological conditions in EAE and nonimmune demyelinated models (18). The immune suppression and anti-inflammatory effects of Bee venom have been reported in MS disease, rheumatoid arthritis and their laboratory models (6). The present study has also indicated results similar to those of the studies on the effects of Bee venom on the inflammatory autoimmune diseases and its anti-inflammatory and immune suppressing activities (6, 8).

TNF-*α* and interferon-γ are the members of pro-inflammatory cytokines, which are mainly secreted by autoimmune T-cells, directly destruct blood-brain barrier and induce apoptosis of oligodendrocytes. These cytokines have also been considered as the demyelination factor (19). Plasma level of TNF-*α* is related to the severity of EAE and MS, which explains the immune status (20).

In the EAE rat model, the serum level of TNF-*α* is increased in the beginning of disease, which shows that TNF-*α* has an important role in the distribution of disease (9).

During the present study, we have observed that TNF-*α* is decreased in a group treated with 5 mg/Kg/day of Bee venom. Bee venom can prevent the production of pro-inflammatory cytokines like TNF-*α* (21).

Nitric oxide (NO) is one of the most important mediators of inflammation during the inflammatory disorders and is produced from the nitric oxide synthase (NOS). There are four members of the NOS family: Neuronal NOS (nNOS), Endothelial NOS (eNOS), Inducible NOS (iNOS), Mitochondrial NOS (Mtons).

iNOS is expressed following the immunological or inflammatory stimulation in macrophages, astrocytes and microglial cells (22). Then is quickly metabolized into nitrite and nitrate. The method of diagnosing nitrite or nitrate in plasma or urine, can be helpful in diagnosing the inflammation process and the treatment of immune disorders (23). Our investigations are in accordance with those of Han *et al.* (2007), who have proven that Bee venom stops the production of NO in microglias, activated by lipopolysaccharides (7). Bee venom acts as an anti-inflammatory agent through the prevention of NOS activity and COX production (24). Bee venom is a preventing factor as it results in the decrease of iNOS activity in Rat C6 glioma cells (25). Cronbach’s alpha value is 76% and R^2^ value is 87%. Data tests confirmed the reliability and validity of this research.

In conclusion, data showed that the treatment of EAE with Bee venom decreases the disease symptoms and pathological changes, level of serum TNF-*α* and nitrate. This activity of Bee venom may be caused by the anti-inflammatory effects and the immuno-modulatory and antioxidant effects of it.

## References

[1] Urbach-Ross D, Kusnecov AW (2007). Effects of acute and repeated exposure to lipopolysacchride on cytokine and corticosterone production during remyelination. Brain Behav. Immun.

[2] Jahangir A, Maftoon F, Sedighi J, Karbakhsh M, Farzadi F, Khodai Sh (2004). The effect of intercessory prayer on quality of life of multiple sclerosis patients. Iranian J. Pharm. Res.

[3] Hedreul MT, Gillett A, Olsson T, Jagodic M, Harris RA (2009). Characterization of multiple sclerosis candidate gene expression kinetics in rat experimental autoimmune encephalomyelitis. J. Neuroimmunol.

[4] Ferguson C, Sarlieve LI, Vincendon G (1990). Multiple sclerosis: review of Main experimental data and pathogenic hypotheses. Revue de Med. Interne.

[5] Mao YS, Lu CZ, Wang X, Xiao BG (2007). Induction of experimental autoimmune encephalomyelitis in Lewis rats by a viral peptide with limited homology to myelin basic protein. Exp. Neurol.

[6] Mirshafiey A (2007). Venom therapy in multiple Sclerosis. Neuropharmacol.

[7] Han SM, Lee KG, Yeo JH, Kweon HY, Woo SK, Lee ML, Baek HJ, Kim SY, Park KK (2007). Effect of honey Bee venom on microglial cells nitric oxide and tumor necrosis factor - α production stimulated by LPS. J. Enthnopharmacol.

[8] Son DJ, Lee JW, Lee YH, Song HS, Lee CK, Hong JT (2007). Therapeutic application of anti-arthritis, pain–releasing, and anti-cancer effects of Bee venom and its constituent compounds. Pharmacol. Ther.

[9] Schnider C, Shuetz G, Zollner TM (2009). Acute neuroinflammation in Lewis rats – A model for acute multiple sclerosis relapses. J. Neuroimmunol.

[10] Okudo Y, Sakoda S, Fujimura H, Saeki Y, Kishimato T, Yanagihara T (1999). IL-6 plays a crucial role in the induction 35-55 induced experimental autoimmune encephalomyelitis. J. Neuroimmunol.

[11] Xia DS, Deng DJ, Wang SL (2003). Destruction of parotid glands affects nitrate and nitrite metabolism. J. Dental Res.

[12] Wang XS, Chen YY, Shang XF, Zhu ZG, Chen GQ, Han Z, Shao B, Yang HM, Xu HQ, Chen JF, Zheng RY (2009). Idazoxan attenuates spinal cord injury by enhanced astrocytic activation and reduced microglial activation in rat experimental autoimmune encephalomyelitis. Brain Res.

[13] Chen GQ, Chen YY, Wang XS, Wu SZ, Yang HM, Xu HQ, He JC, Wang XT, Chen JF, Zheng RY (2010). Chronic caffeine treatment attenuates experimental autoimmune encephalomyelitis induced by guinea pig spinal cord homogenates in Wistar rats. Brain Res.

[14] Kwon YB, Lee HJ, Han HJ, Mar WC, Kang SK, Yoon OB, Beitz AJ, Lee JH (2002). The water soluble fraction of Bee venom produces antinociceptive and anti- inflammatory effects on rheumatoid arthritis in rats. Life Sci.

[15] Jutel M, Akdis M, Blaser K (2005). Are regulatory T cells the target at venom immunotherapy? Curr. Opn. Allergy Clin. Immunol.

[16] Hamedani M, Vatanpour H, Saadat F, Khorramizadeh MR, Mirshafiey A (2005). Bee venom immunostimulant or immune suppressor? Insight into the effect on matrix metalloproteinases and interferons. Immunopharmacol. Immunotoxicol.

[17] Dong M, Liu R, Guo L, Li C, Tan G (2008). Pathological finding in rats with experimental allergic encephalomyelitis. APMIS.

[18] Mastronardi FG, Min W, Wang H, Winer SH, Dosch M, Boggs JM, Moscarello MA (2004). Attenuation of experimental autoimmune encephalomyelitis and nonimmune demyelination by IFN–ß plus vitamin B12: Treatment to modify notch –1 / sonic hedgehog balance. J. Immunol.

[19] Akassoglu K, Bauer G, Kassiatis M (1998). Olligodendrocyte apoptosis and primary demyelination induced by local TNF/P55TNF receptor signaling in the central nervous system of transgenic mice: models for multiple sclerosis with primary oligodenrogliopathy. Am. J. Pathol.

[20] Pollak Y, Ovadia H, Orion E, Weidenfeld J, Yirmiya R (2003). The EAE-associated behavioral syndrome: I Temporal correlation with inflammatory mediators. J. Neuroimmunol.

[21] Nam KW, Je KH, Lee JH, Han HJ, Lee HJ, Kang SK, Mar W (2003). Inhibition of cox-2 activity and proinflammatory cytokines (TNF-alpha and IL-1beta) production by water–soluble sub–fractionated parts from Bee (Apis mellifera) venom. Arch. Pharmacol. Res.

[22] Guix FX, Uribesalgo I, Coma M, Munoz FJ (2005). The physiology and pathophysiology of nitric oxide in brain. Progr. Neurobiol.

[23] Moshage E (1997). Nitric oxide determination: much ado about nothing? Clin. Chem.

[24] Jang HS, Kim SK, Han JB, Ahn HJ, Bae H, Min BI (2005). Effect of Bee venom on the pro-inflammatory responses in RAW 264.7 macrophage cell line. J. Ethnopharmacol.

[25] Lee KG, Cho HJ, Bae YS, Park KK, Choe JU, Chung IL, Kim M, Yeo JH, Park KH, Lee YS (2009). Bee venom suppresses LPS–Mediated NO/iNOS induction through inhibition of PKC- expression. J. Enthnopharmacol.

